# Supplementary material for: Safety and Immunogenicity of the Third Booster Dose with Inactivated, Viral Vector, and mRNA COVID-19 Vaccines in Fully Immunized Healthy Adults with Inactivated Vaccine
Source: Vaccines (Basel). 2022 Jan 6;10(1):86. doi: 10.3390/vaccines10010086 (PMC8779615; doi:10.3390/vaccines10010086)
Supplement: Supplementary file 1 [file vaccines-10-00086-s001.zip › vaccines-1516362-supplementary.pdf]

Supplementary information

Supplementary Figure and Tables

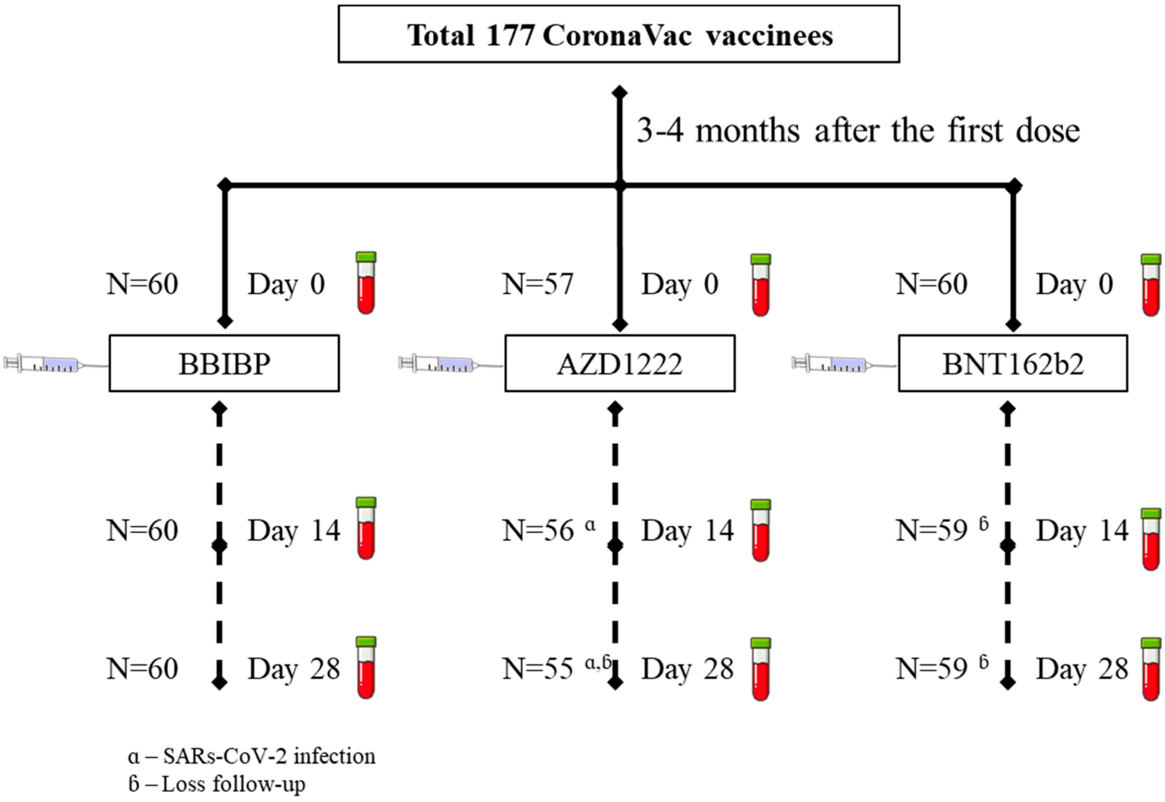

Supplementary Figure S1. The participant flow diagram of this clinical study of third booster and blood sampling collection.

Supplementary Table S1 Statistic analysis of reactogenicity data of between the booster vaccines.

The Fisher's Exact Test was used to interpret the statistical analysis.

	**BBIB**P	**AZD1222**	***p* value**	**Result**	**BBIB**P	**BNT162b2**	***p* value**	Result	**AZD1222**	**BNT162b2**	***p* value**	**Result**
**n**												
**Total (%)**	60 (100.00)	57 (100.00)			60 (100.00)	60 (100.00)			57 (100.00)	60 (100.00)		
**Injection site pain**	33 (55.00)	52 (91.23)	< 0.0001	Yes	33 (55.00)	59 (98.33)	< 0.0001	Yes	52 (91.23)	59 (98.33)	0.1079	No
**Swelling**	6 (10.00)	25 (43.86)	< 0.0001	Yes	6 (10.00)	25 (41.67)	0.0001	Yes	25 (43.86)	25 (41.67)	0.8531	No
**Redness**	4 (6.67)	18 (31.58)	0.0007	Yes	4 (6.67)	21 (35.00)	0.0002	Yes	18 (31.58)	21 (35.00)	0.8446	No
**Fever**	0 (0.00)	9 (15.79)	< 0.0001	Yes	0 (0.00)	5 (8.33)	0.0573	No	9 (15.79)	5 (8.33)	0.2615	No
**Headache**	10 (16.67)	41 (71.93)	< 0.0001	Yes	10 (16.67)	34 (56.67)	< 0.0001	Yes	41 (71.93)	34 (56.67)	0.1226	No
**Myalgia**	19 (31.67)	44 (77.19)	< 0.0001	Yes	19 (31.67)	44 (73.33)	< 0.0001	Yes	44 (77.19)	44 (73.33)	0.6729	No
**Nausea**	2 (3.33)	10 (17.54)	0.0142	Yes	2 (3.33)	11 (18.33)	0.0159	Yes	10 (17.54)	11 (18.33)	>0.9999	No
**Vomiting**	0 (0.00)	3 (5.26)	0.1125	No	0 (0.00)	5 (8.33)	0.0573	No	3 (5.26)	5 (8.33)	0.7175	No
**Diarrhea**	5 (8.33)	16 (28.07)	0.0074	Yes	5 (8.33)	10 (16.67)	0.2693	No	16 (28.07)	10 (16.67)	0.1825	No
**Joint pain**	3 (5.00)	23 (40.35)	< 0.0001	Yes	3 (5.00)	13 (21.67)	0.0136	Yes	23 (40.35)	13 (21.67)	0.0443	Yes
**Chilling**	5 (8.33)	32 (56.14)	< 0.0001	Yes	5 (8.33)	27 (45.00)	< 0.0001	Yes	32 (56.14)	27 (45.00)	0.2690	No
**Dizziness**	7 (11.67)	21 (36.84)	0.0021	Yes	7 (11.67)	23 (38.33)	0.0013	Yes	21 (36.84)	23 (38.33)	>0.9999	No

Supplementary Table S2. The data values from laboratory testing of participants who received the booster dose with BBIBP, AZD122 or BNT162b2, which referred to graphic info displays.

	BBIBP	AZD1222	BNT162b2
**Humoral responses**			
**Ig anti-RBD (U/mL)**			
Day 0 (baseline), n<br>GMT (95% CI)	60<br>35.45 (25.8 - 48.71)	57<br>37.89 (27.39 - 52.41)	60<br>48.57 (36.54 - 64.58)
Day 14, n<br>GMT (95% CI)	60<br>1,073 (849.4 - 1,355)	56<br>9,865 (7,990 - 12,182)	59<br>20,787 (18,229 - 23,703)
Day 28, n<br>GMT (95% CI)	60<br>839.9 (674.1 - 1,047)	55<br>8,160 (6,635-10,035)	59<br>13,871 (11,993 - 16,043)
**IgG anti-RBD (BAU/mL)**			
Day 0 (baseline), n<br>GMT (95% CI)	60<br>42.76 (33.78 - 54.12)	57<br>41.13 (33.22 - 50.93)	60<br>48.99 (40.77 - 58.88)
Day 14, n<br>GMT (95% CI)	60<br>205.5 (167.0 - 253.0)	56<br>1,936 (1,597 - 2,346)	59<br>3,821 (3,306 - 4,416)
Day 28, n<br>GMT (95% CI)	60<br>164.1 (133.8 - 201.1)	55<br>1,736 (1,434 - 2,101)	59<br>2,584 (2,250 - 2,966)
**IgG anti-N index (S/C)**			
Day 0 (baseline), n<br>Median (IQR)	60<br>0.265 (0.140 - 1.130)	57<br>0.230 (0.095 - 0.810)	60<br>0.535 (0.170 - 1.078)
Day 14, n<br>Median (IQR)	60<br>2.970 (1.715 - 4.745)	56<br>0.205 (0.083 - 0.615)	59<br>0.51 (0.180 - 0.880)
Day 28, n<br>Median (IQR)	60<br>2.780 (1.513 - 4.643)	55<br>0.170 (0.070 - 0.400)	59<br>0.51 (0.230 - 0.880)
**IgA anti-S1 ratio (S/C)**			
Day 0 (baseline), n<br>Median (IQR)	20<br>0.685 (0.453 - 0.845)	20<br>0.455 (0.255 - 0.628)	20<br>0.675 (0.453 - 0.845)
Day 14, n<br>Median (IQR)	60<br>2.055 (1.073 - 4.383)	56<br>8.515 (5.23 – 9.00)	59<br>9.00 (9.00 - 9.00)
Day 28, n<br>Median (IQR)	60<br>1.300 (0.565 - 2.955)	55<br>7.450 (4.09 – 9.00)	59<br>9.00 (7.12– 9.00)
**Neutralization assay**<br>**sVNT-WT (%inhibition)**			
Day 0 (baseline), n<br>Median (IQR)	10<br>20.9 (18.33 - 36.95)	10<br>35.15 (19.23 - 46.45)	10<br>40.25 (20.48 - 45.93)
Day 14, n<br>Median (IQR)	N/D	N/D	N/D
Day 28, n<br>Median (IQR)	30<br>79.75 (62.33 - 89.93)	30<br>97.60 (97.20 - 97.80)	30<br>97.80 (97.68 - 97.90)
**Neutralization assay**<br>**sVNT-Alpha (%inhibition)**			
Day 0 (baseline), n<br>Median (IQR)	10<br>9.25 (4.50 - 18.53)	10<br>17.30 (17.30 - 26.08)	10<br>19.45 (12.15 - 25.03)
Day 14, n<br>Median (IQR)	N/D	N/D	N/D
Day 28, n<br>Median (IQR)	30<br>67.20 (51.10 - 81.80)	30<br>96.50 (94.58 - 97.33)	30<br>97.05 (97.05 - 97.53)
**Neutralization assay**<br>**sVNT-Beta (%inhibition)**			
Day 0 (baseline), n<br>Median (IQR)	10<br>-3.80 ((-8.28) - 2.50)	10<br>-0.15 ((-19.93) - 10.68)	10<br>1.15 ((-10.95) - 7.50)

Day 14, n<br>Median (IQR)	N/D	N/D	N/D
Day 28, n<br>Median (IQR)	30<br>50.55 (36.35 - 67.18)	30<br>91.85 (87.80 - 93.98)	30<br>93.30 (90.65 - 94.90)
**Neutralization assay**<br>**sVNT-Delta (%inhibition)**			
Day 0 (baseline), n<br>Median (IQR)	10<br>24.40 (17.98 - 30.13)	10<br>30.95 (20.70 - 42.65)	10<br>27.40 (20.15 - 40.43)
Day 14, n<br>Median (IQR)	N/D	N/D	N/D
Day 28, n<br>Median (IQR)	30<br>72.00 (53.75 - 86.93)	30<br>97.35 (96.45 - 97.73)	30<br>97.60 (97.18 - 97.80)
**T-cell responses**<br>**IFN-γ $CD4^+$ (IU/mL)**			
Day 0 (baseline), n<br>Median (IQR)	60<br>0.050 (0.020 - 0.140)	57<br>0.030 (0.000 - 0.145)	60<br>0.035 (0.000 – 0.128)
Day 14, n<br>Median (IQR)	60<br>0.125 (0.053 - 0.453)	56<br>0.500 (0.188 - 1.173)	58*<br>1.250 (0.430 - 3.698)
Day 28, n<br>Median (IQR)	60<br>0.085 (0.030 - 0.238)	55<br>0.260 (0.090 - 0.710)	59<br>0.790 (0.290 - 1.890)
**IFN-γ $CD4^+$ $CD8^+$ (IU/mL)**			
Day 0 (baseline), n<br>Median (IQR)	60<br>0.075 (0.030 - 0.258)	57<br>0.040 (0.010 - 0.210)	60<br>0.050 (0.010 - 0.208)
Day 14, n<br>Median (IQR)	60<br>0.240 (0.060 - 0.615)	56<br>0.720 (0.325 - 1.690)	58*<br>2.020 (0.538 - 4.993)
Day 28, n<br>Median (IQR)	60<br>0.155 (0.053 - 0.410)	55<br>0.470 (0.170 - 1.430)	59<br>1.260 (0.400 - 3.090)

N/D – do not determine.

* – one participant's sample is not adequate to evaluate QFN assay because we were unable to obtain the appropriate amount of heparinized blood.
